# Frailty in Secure Forensic Mental Health Settings: A Study from Dundrum Hospital, Ireland

**DOI:** 10.1192/j.eurpsy.2022.885

**Published:** 2022-09-01

**Authors:** F. Murphy, A. Mcloughlin, A. Butler, M. Davoren, H. Kennedy

**Affiliations:** 1 Central Mental Hospital, National Forensic Mental Health Service, Dundrum, Dublin, Ireland; 2 Beaumont Hospital, Department Of Psychiatry Of Later Life, Dublin, Ireland; 3 Department of Psychiatry, Trinity College Dublin, Dublin, Ireland

**Keywords:** Frailty, psychiatry, comorbidity, forensic

## Abstract

**Introduction:**

Frailty is defined as a clinical syndrome that encompasses a combination of decreased physiological reserve and low resistance to stressors. There is an association between mental illness and frailty among elderly cohorts. Frailty is also associated with obesity and smoking. There are high rates of treatment resistant schizophrenia among patients in secure forensic services. Patients with schizophrenia have high rates of morbidity and early mortality.

**Objectives:**

The primary aim of this study was to examine the rates of frailty present in a complete cohort of forensic in-patients.

**Methods:**

An assessment using Fried Frailty criteria was offered to all in-patients (n=95) in Ireland’s National Forensic Service, which included measures of walking speed, grip strength, low physical activity and exhaustion. Demographic details and details pertaining to diagnoses and medications were also gathered.

**Results:**

Of the 95 in-patients, 92 patients agreed to participate. The majority were male (89%). The most common diagnosis was schizophrenia (71.7%). Mean age was 44.7 years (SD 11.42), and 58.2% met criteria for obesity. Of the total group, 47 patients met criteria for ‘pre-frail’ and 10 met criteria for ‘frail’ using Fried criteria.

**Conclusions:**

This is the first study examining frailty in a cohort of patients in secure forensic settings. We found high rates of patients meeting frailty criteria at very young ages. Rates of frailty in this group were comparable to those found amongst elders in community settings. We consider this demonstrates significant medical vulnerability in this patient group.

**Disclosure:**

No significant relationships.

